# The Flower Extract of *Abelmoschus manihot* (Linn.) Increases Cyclin D1 Expression and Activates Cell Proliferation

**DOI:** 10.4014/jmb.2002.02024

**Published:** 2020-03-09

**Authors:** Yea-In Park, Yeo-Eun Cha, Minsu Jang, Rackhyun Park, Sim Namkoong, Jongbock Kwak, Ik-Soon Jang, Junsoo Park

**Affiliations:** 1 Division of Biological Science and Technology, Yonsei University, Wonju 26493, Republic of Korea; 2 Department of Biochemistry, Kangwon National University, Chuncheon 24341, Republic of Korea; 3 ALL IN ON, Seoul 06173, Republic of Korea; 4 Division of Analytical Science, Korea Basic Science Institute, Daejeon 34133, Republic of Korea

**Keywords:** *Amelmoschus manihot*, flower extract, cell proliferation, cyclin D1

## Abstract

*Abelmoschus manihot* (Linn.) is a medicinal herbal plant that is commonly used to treat chronic kidney disease and hepatitis. However, its effect on cell proliferation has not been clearly revealed. In this report, we sought to determine the effect of the flower extract of *A. manihot* (FA) on cell proliferation. Based on our findings, FA increased the proliferation of human diploid fibroblast (HDF) and HEK293 cells. Through cell cycle analysis, FA was found to increase the number of HDF cells in the S phase and G2/M phase. FA also increased the expression of cyclin D1 and enhanced the migration of HDF cells. By administering FA to HDF cells with ≥ 30 passages, a decrease in the number of senescence-associated β galactosidase-positive cells was observed, thereby indicating that FA can ameliorate cellular senescence. Collectively, our findings indicate that FA increases cyclin D1 expression and regulates cell proliferation.

## Introduction

Medicinal plants are traditionally used to treat different diseases. One of the medicinal plants, *Abelmoschus manihot* (Linn), belongs to the Malvaceae family and is used to treat many diseases [1, 2]. The flower of *A. manihot*, its main medicinal part, exerts anti-inflammatory, anti-oxidant, and anti-coagulation activities, and is thus used to treat chronic kidney disease, diabetic nephropathy, oral ulcer, and burns [1,3-8]. Previously, the flavonoids from the *A. manihot* flowers were demonstrated to exhibit an inhibitory effect on the accumulation of triglyceride in 3T3-L1 cells and anticonvulsant and antidepressant effects in mice [9, 10]. In addition, they exerted cardioprotective effects on myocardial ischemia and reperfusion in animal experiments [11], and high performance liquid chromatography (HPLC) analysis revealed flavonoids, such as quercetin and myricetin, in the flowers [12, 13].

Cyclins are well known as critical regulators of the cell cycle. In fact, cyclin D-cdk4 or cdk6 activation promotes cell cycle progression via the phosphorylation of the RB protein [14]. The overexpression of cyclin D1 and its nuclear retention contribute to cell proliferation [15]. Cyclin D1 expression also promotes cell migration and wound healing [16, 17]. Therefore, the expression level of cyclin D1 is important for wound healing.

In this study, we examined the effect of the flower extract of *A. manihot* (FA) on cell proliferation and migration. By using 1,3-butylene glycol for FA extraction, we found that FA enhanced cell proliferation by regulating the cell cycle. These results support the potential use of FA as a treatment option for diseases.

## Materials and methods

### Plant Materials

FA was provided by ALL IN ON (Seoul, Korea). Briefly, the fresh flowers of *A. manihot* were collected, washed, and dried in shade. The dried flowers (50 g) were chopped into pieces and suspended in 1, 3-butylene glycol solution (40%, 500 g) for 24 h at room temperature. The mixture was filtered with a mesh filter and membrane filters (3 μm and 1 μm, sequentially). Finally, the filtrate was filtered again with a membrane filter (0.45 μm) to remove microorganisms. The final filtrate was dried and suspended with DMSO.

### Cell Culture and Cell Proliferation Assay

Human diploid fibroblast (HDF) cells and HEK293 cells were grown in Dulbecco’s Modified Eagle’s medium (Welgene, Korea) supplemented with 10% fetal bovine serum (Gibco, USA). High passage HDF cells are defined as HDF cells at passage ≥30. Cell proliferation was measured with an EZ-cytox cell viability/cytotoxicity kit (Daeillab service, Korea). Briefly, equal numbers of cells were seeded in the wells of a 24-well plate. At the indicated time, the EZ-cytox solution was added to the cells; thereafter, the cells were incubated according to the manufacturer’s protocol. Finally, the absorbance of the media was measured at 450 nm. For cell cycle analysis, cells were washed with phosphate buffered saline (PBS) and fixed with 70% ethanol. After centrifugation, cells were washed and resuspended in PBS containing 50 μg/ml propidium iodide and 10 mg/ml RNase A. FA-treated cells were analyzed with a FACSCalibur flow cytometer (Beckton-Dickson, USA).

### Western Blot

For western blot analysis, polypeptides in whole cell lysates were resolved by SDS-PAGE and transferred to a nitrocellulose membrane. Detection was carried out with a 1:5,000 or 1:10,000 dilution of the primary antibody using an enhanced chemiluminescence (ECL) system. Images were acquired with an LAS4000 system (GE Healthcare, Sweden). The antibodies for cyclin D1, phospho Erk 1/2 and Erk 1/2 were purchased from Cell Signaling Technology (USA).

### Immunofluorescence Staining and Confocal Microscopy

Cells were grown on sterilized glass coverslips. After drug treatment, the cells were fixed with 4% paraformaldehyde. Thereafter, the cells were blocked with 10% goat serum in PBS, stained with a 1:500 dilution of primary antibody in PBS, and then reacted with a 1:1,000 dilution of Alexa 488- or Alexa 568-conjugated secondary antibody (Invitrogen) for immunostaining. Finally, the slides were washed three times with PBS, stained with DAPI, and mounted in mounting medium (Vector, USA). Images were captured with a Carl Zeiss LSM710 confocal microscope (Carl Zeiss, Germany).

### Senescence-Associated β Galactosidase (SA-β gal) Staining

We employed the SA-β gal staining kit (Cell Biolabs, USA) for SA-β gal staining. Briefly, 10,000 HDF cells were plated on a 6-well plate. After treatment with FA, cells were fixed and stained with SA-β gal, according to the manufacturer’s protocols. After staining, images were captured with an Olympus BX53 microscope (Japan). The number of SA-β gal-positive cells was counted for further analysis.

### LC-MS Apparatus and Conditions for Quantitative Analysis of FA

Agilent 6410B Triple Quadrupole LC/MS (Agilent Technologies, USA) equipped with an ESI source was employed for the analysis. Aliquots of 5 μl of the processed samples were injected into the high-performance liquid chromatography system (1200 Series LC; Agilent Technologies,) fitted with a Synergi Hydro-RP 4 μm 80 Å 150 × 2 mm column (Phenomenex, USA) maintained at 30oC. ESI was operating at + 3,000 V and the source temperature of 380oC. Data acquisition was performed using MassHunter Software Version B.04.00 (Agilent Technology).

### Statistical Analysis

The results of the western blot analysis, cell proliferation assay (EZ-Cytox, MTT) and SA-β gal assay were evaluated by a 2-tailed *t* test using Excel software (Microsoft, USA). *p* < 0.05 was considered significant.

## Results

### FA Enhances Cell Proliferation

**Fig. 1:**
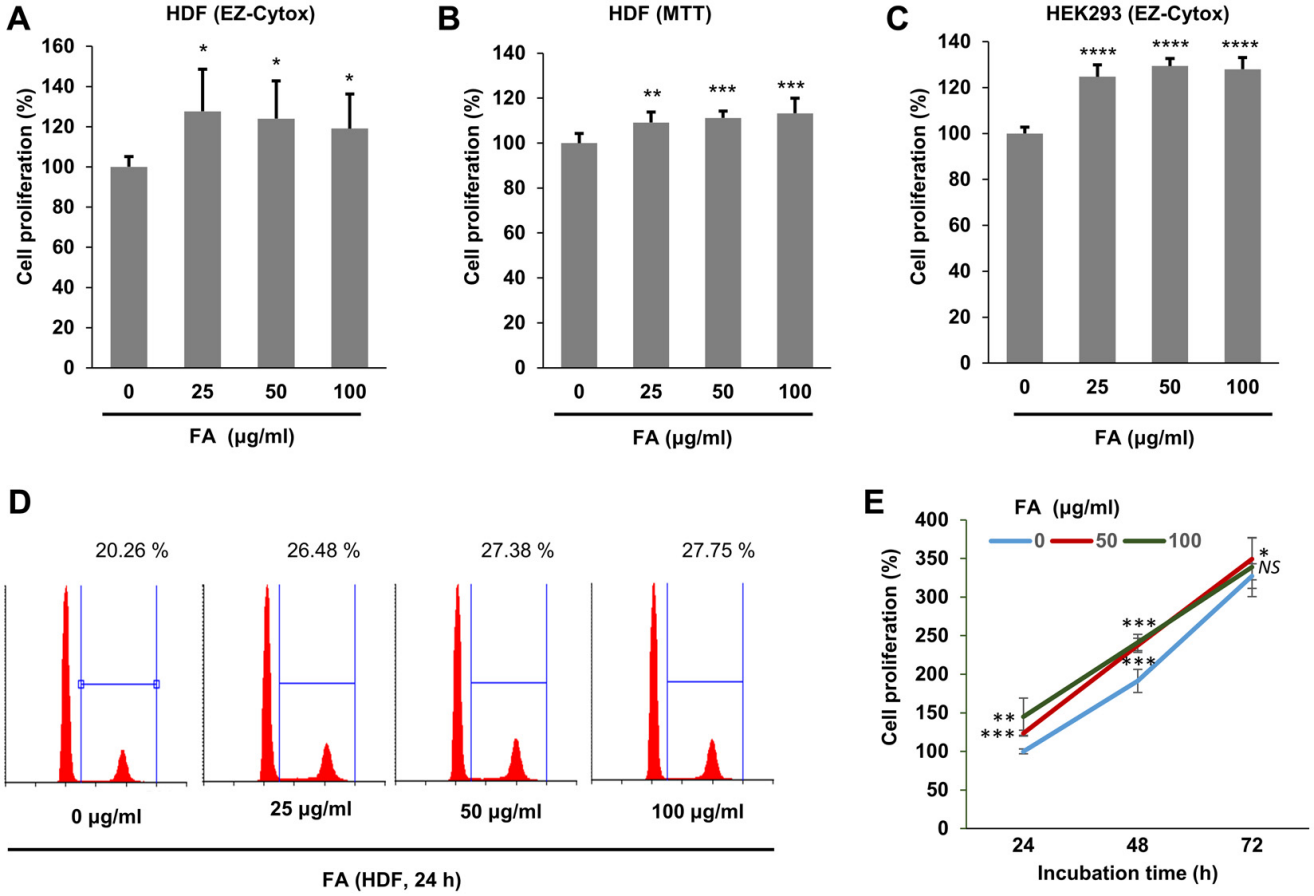
FA increased the proliferation of HDF cells. (**A**) HDF cells were incubated with the indicated concentration of the FA extract for 24 h, and cell proliferation was evaluated with an EZ-Cytox cell proliferation assay kit (N = 8). The graph shows the average and standard deviation. Mock vs FA treatment (N = 8), *p <0.05, *NS* not significant. (**B**) Cell proliferation was evaluated using the MTT cell proliferation assay kit. Mock vs FA treatment (N = 8), **p < 0.005, ***p < 0.001. (**C**) HEK293 cells were incubated with the FA extract, and cell proliferation was evaluated at the indicated time (N = 8). ****p < 0.0001. (**D**) HDF cells were incubated with the FA extract for 24 h, and the cells were stained with propidium iodide and analyzed by flow cytometry. The numbers indicate the proportion of cells in the S phase and G2/M phase. (E) HDF cells were treated with the FA extract, and cell proliferation was evaluated at the indicated time. Mock vs FA treatment (N = 8), *p < 0.05, **p < 0.005, ***p < 0.0005. *NS* means not significant.

We attempted to evaluate the biological effects of FA. Initially, we examined the effect of FA on cell proliferation using normal HDF cells. HDF cells were incubated with FA, and cell proliferation was evaluated using two different cell viability assay kits. FA significantly increased the cell proliferation of HDF cells (Figs. 1A and 1B). By administering FA to HEK293 cells, FA was found to increase cell proliferation (Fig. 1C). In addition, we examined the cell cycle of HDF cells following treatment with FA. Based on our findings, FA increased the proportion of cells in the S phase and the G2/M phase (Fig. 1D). In addition, the proportion of the sub-G1 population was not increased by FA, indicating that FA did not induce apoptosis. To examine the potential toxicity of FA, we administered FA to HDF cells for an extended time (48 h and 72 h). However, FA did not decrease the cell proliferation for 48 h and 72 h, indicating that FA did not exhibit any toxicity during long incubation periods. These results collectively indicate that FA increases cell proliferation.

### FA Increases Cyclin D1 Expression

**Fig. 2:**
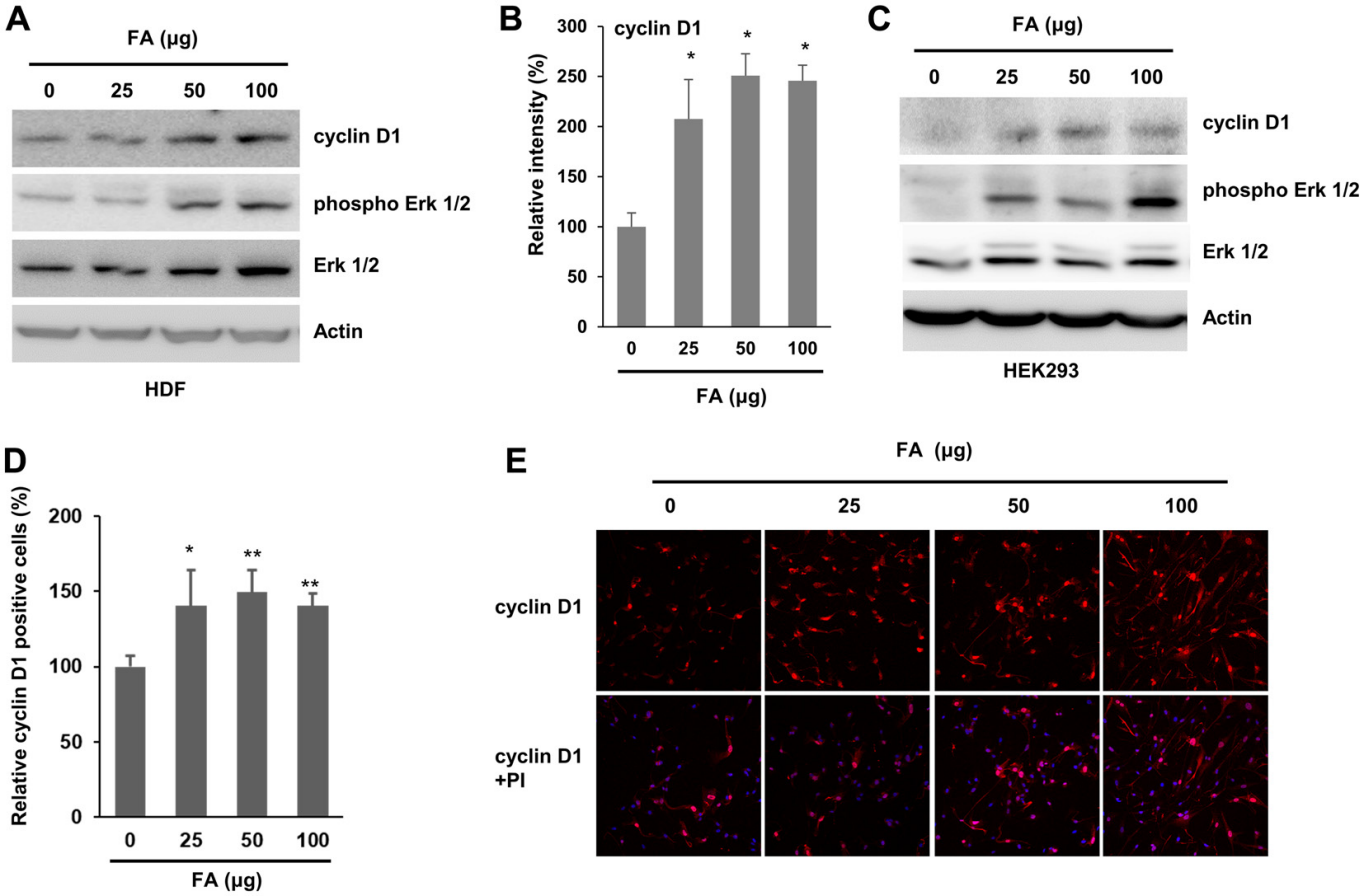
FA increased the expression of cyclin D1. (**A**) HDF cells were incubated with the indicated concentration of FA. After 24 h, the cells were lysed and an equal amount of cell lysate was subjected to western blot with the indicated antibodies. (**B**) The level of cyclin D1 was evaluated and is depicted in the graph. The graph shows the average and standard error. Mock vs FA treatment. *p < 0.05. (**C**) The level of cyclin D1 was evaluated in HEK293 cells upon FA treatment. (**D**, **E**) HDF cells were incubated with FA, and the cells were immunostained with the anti-cyclin D1 antibody. The number of cyclin D1-positive cells was analyzed and is depicted in the graph. *p < 0.05, **p < 0.05.

Because FA increases the proportion of cells in the S phase and G2/M phase, we examined the level of cyclin D1, an important cell cycle regulator. As expected, the level of cyclin D1 increased upon FA (Figs. 2A and 2B). Additionally, the level of phospho Erk increased following treatment with FA (Fig. 2A). Together, these results indicate that FA enhances the cell cycle by regulating cyclin D1 expression. When cyclin D1 expression level was examined using HEK293 cells, similar results were obtained (Fig. 2C). HDF cells were stained with FA using the cyclin D1 antibody. Based on our findings, the proportion of cyclin D1-positive cells was increased by treatment with FA (Figs. 2D and 2E), indicating that cyclin D1 expression is increased by FA.

### FA Did Not Affect NF-κB Activity

**Fig. 3:**
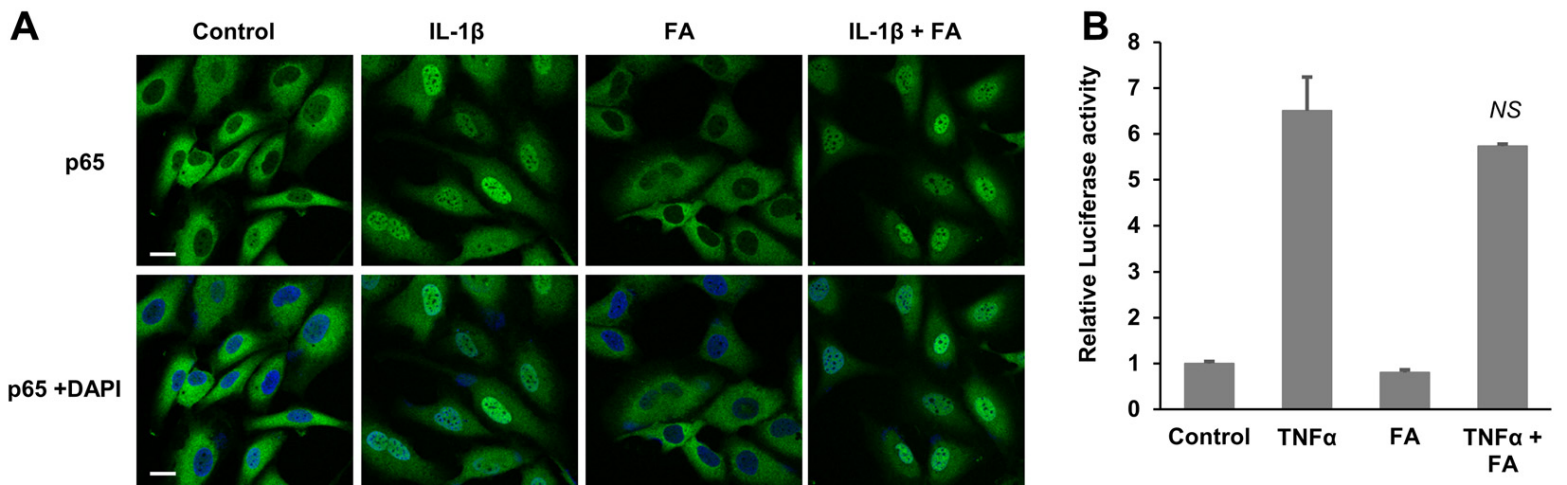
FA did not affect NF-κB signaling. (**A**) SK-OV3 cells were treated with IL-1β in the absence or presence of the indicated concentration of FA. The cells were fixed and stained with the anti-p65 (RelA) antibody. (**B**) HEK293 cells were transfected with the reporter plasmid, pELAM-Luc, and treated with TNFα and/or FA for 24 h. The graph shows the average and standard deviation. *NS*, not significant.

Because the total flavone of *A. manihot* decreased NF-κB activity [18], we determined whether FA affects NF- κB activity. First, we examined the location of p65 (RelA) upon FA treatment. Normally, p65 is localized in the cytoplasm and is transported to the nucleus by IL-1β (Fig. 3A). However, FA did not affect the subcellular localization of p65 in the presence or absence of IL-1β (Fig. 3A). These findings suggest that FA did not affect NF- κB activity. As a result, we proceeded to examine NF-κB activity by using a luciferase reporter containing an NF- κB-dependent promoter. HEK293 cells were transfected with a pELAM-Luciferase construct and treated with TNFα in the presence or absence of FA. Although TNFα increased NF-κB activity, FA did not affect this activity (Fig. 3B). These findings indicate that FA does not significantly affect NF-κB activity.

### FA Enhances Cell Migration

**Fig. 4:**
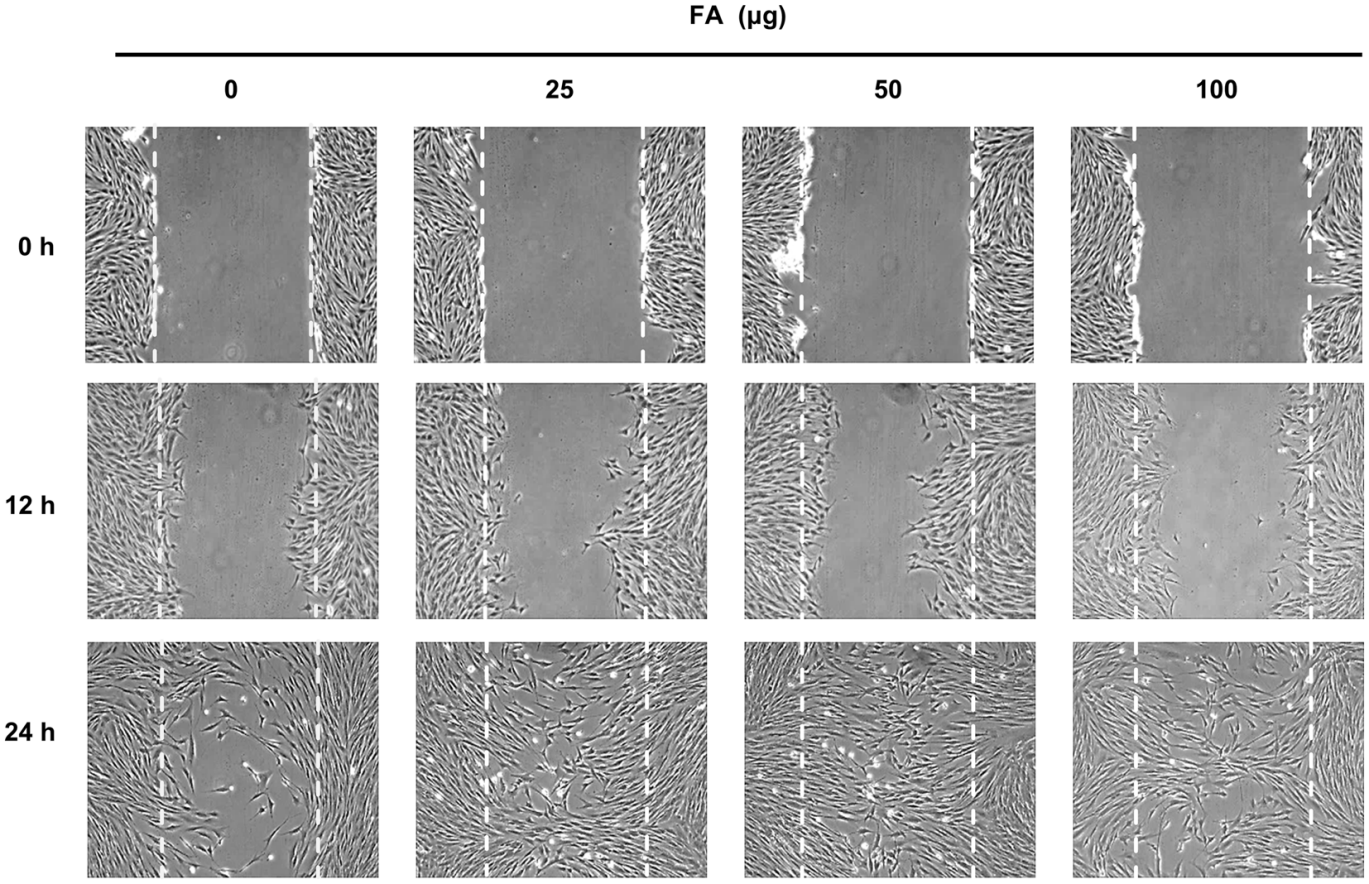
FA activated cell migration. Treatment with FA activated the migration of HDF cells. Equal numbers of HDF cells were plated onto a 6-well plate, and cells were scraped with a pipette tip. After the indicated times, images of the wound gaps were captured with an inverted microscope.

Previous reports revealed that cyclin D regulates cell migration by regulating ROCK signaling [16]. Because FA increases the expression of cyclin D1, we hypothesized that FA affects cell migration. To test our hypothesis, we examined the effect of FA on cell migration by performing a wound healing assay, which can be used to examine cell migration and cell-cell interaction. By using HDF cells, we found that FA evidently enhanced cell migration at 25, 50, and 100 μM (Fig. 4). Such findings indicate that treatment with FA activates cell migration.

### FA Decreases SA-β gal

**Fig. 5:**
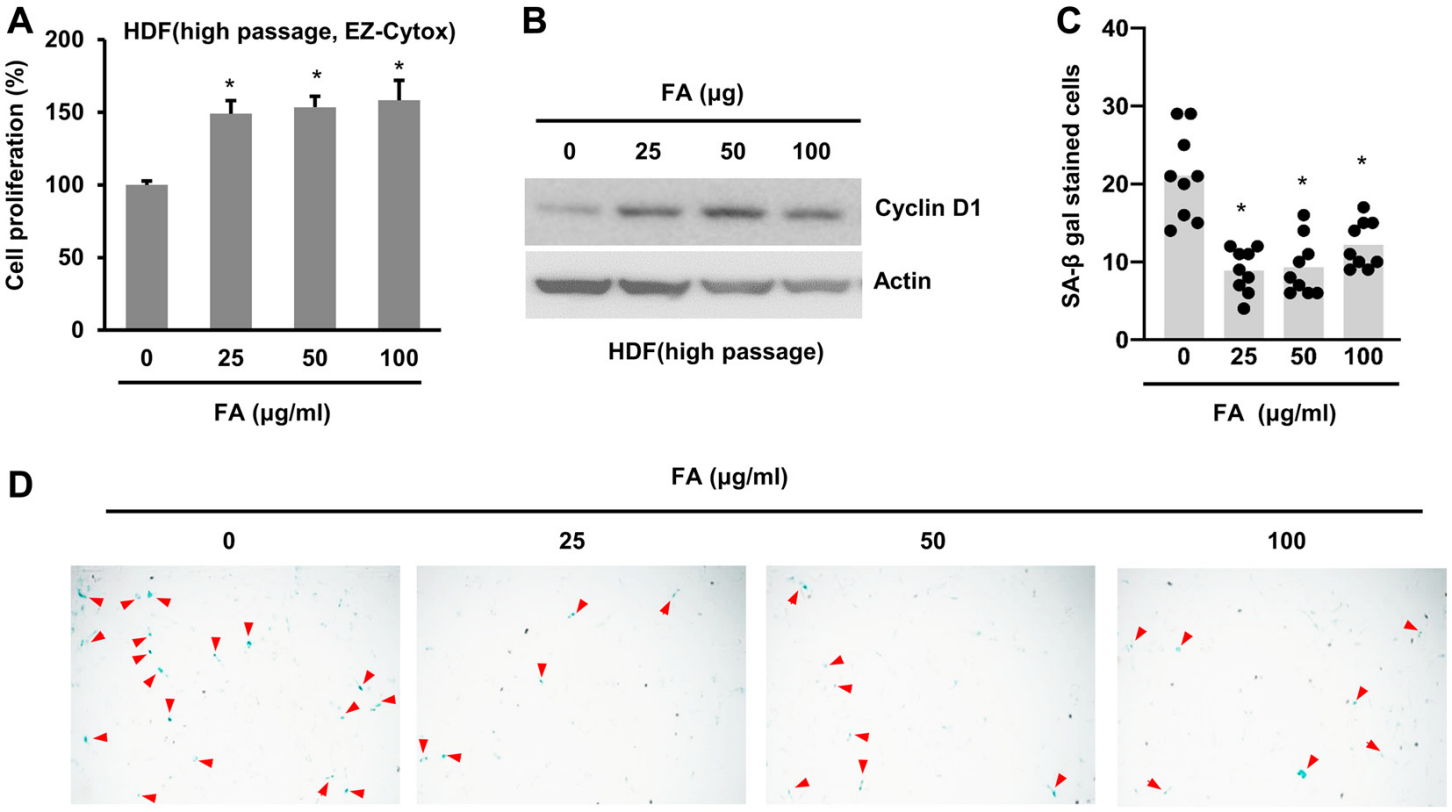
FA reduced the number of SA-β gal-positive cells. (**A**) High passage HDF cells (passage number ≥30) were incubated with FA, and cell proliferation was analyzed with the EZ-Cytox cell proliferation assay kit. Mock vs FA treatment. *p < 0.001. (**B**) The level of cyclin D1 was evaluated by western blot. (**C**, **D**) High passage HDF cells were stained with the SA-β gal staining kit. Mock vs FA treatment. *p < 0.001.

Because FA activates cell proliferation, we anticipated the proliferation of senescent cells. As a result, we used a high passage of HDF cells (high passage HDF; *i.e*., passage number, *≥*30). These HDF cells were found to contain senescent cells [19]. First, we administered FA to high passage HDF cells and examined cell proliferation. As expected, FA increased the proliferation of the high passage HDF cells (Fig. 5A). Thereafter, we examined the expression level of cyclin D1 which was found to be elevated in high passage HDF cells (Fig. 5B).

We proceeded to examine whether FA affects cellular senescence. The positive staining of SA-β gal is known to indicate senescence; thus, high passage HDF cells can be stained with SA-β gal [19]. To examine whether FA affects senescence, we administered FA to high passage HDF cells and counted the number of SA-β gal-positive cells. Based on our findings, FA decreased the number of SA-β gal-positive cells, indicating that FA could decrease the number of senescent cells (Figs. 3C and 3D). Altogether, our results indicate that FA ameliorates cellular senescence.

### LC-MS Analysis of FA

We proceeded to analyze the FA composition by liquid chromatography mass spectroscopy (LC-MS) analysis and found that it contained myricetin, quercetin, and cannabiscitrin, which were previously reported [12, 13]. FA was also found to contain many active components, such as quinolone, morin, and kaempferol. Collectively, these findings indicate that FA contains many active flavonoids.

## Discussion

In the present study, we investigated the effect of FA on cell proliferation. FA increased cell proliferation by regulating the cell cycle. In addition, the expression level of the cyclin D1 protein was increased by treatment with FA. Although we anticipated that FA would decrease NF-κB activity, treatment with FA did not affect this activity. However, we found that it increased cellular migration and decreased the number of SA-β gal-positive cells. These findings suggest that the FA is potentially useful for the treatment of wounds via its activation of cellular activity.

Many cell proliferative signals activate the expression of cyclin D1. For example, Ras signaling, Wnt signaling, and integrin signaling can activate the expression of cyclin D1. In this study, we revealed that FA can activate Erk signaling, which is downstream of Ras signaling. Although the signaling mechanism of cyclin D1 activation should be further investigated, FA activated the cell proliferative signaling pathways and induced cell proliferation by increasing cyclin D1 expression.

The flowers of *Abelmoschus manihot* have been used to treat ulcers and burns. Treatments for these diseases require cell proliferation and cell migration. In this study, we demonstrated that FA affects cell proliferation and migration, which are activities that will contribute to the treatment of ulcers and burns. The activation of cell proliferation by FA will also enable the application of FA in other diseases that require cell proliferation.

Interestingly, FA ameliorated the cellular senescence of high passage HDF cells. Senescent cells had reduced cell proliferation ability, and FA increased their cell proliferation rate. Further, FA decreased the senescence- associated β-galactosidase staining. These findings suggest that FA could be an effective agent for treating old cells. Because FA activates the proliferation of old cells, it can be used as a wound healing agent for old tissues, such as the skin. Further study will be required to reveal the effect of FA in vivo.

## Figures and Tables

**Fig. 6:**
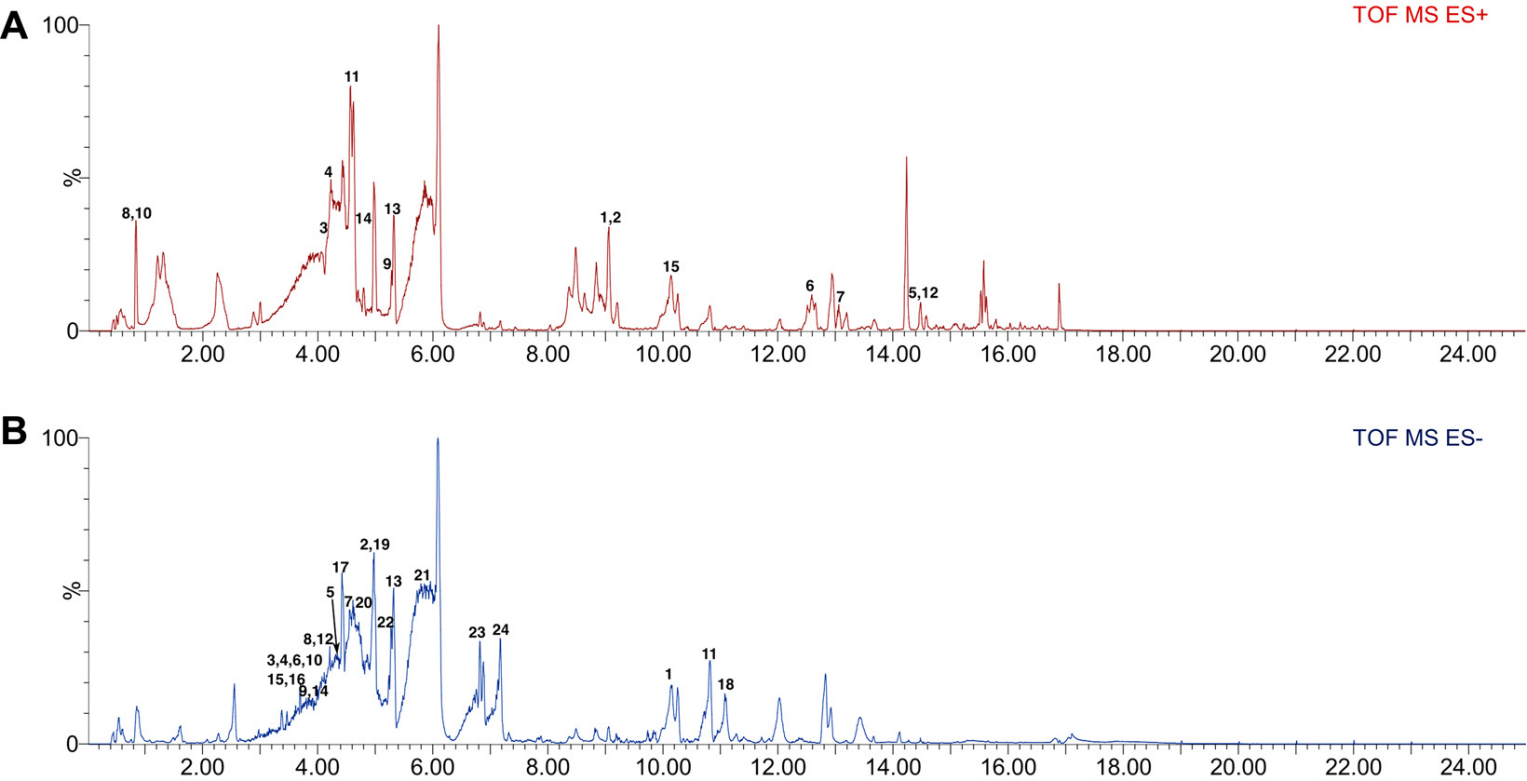
LC-MS analysis of FA. FA was analyzed with a QTof mass spectrometer. Two ionization modes were used (ES positive, ES negative) for the MS analysis. (**A**) ES positive: (1,2) 1-Methyl-2-[(Z)-8-tetradecenyl]-4(1H)-quinolone, (3) 1- Methyl-2-[(Z)-8-tetradecenyl]-4(1H)-quinolone, (4) 5,7,8,3′,4′-Pentamethoxy flavone, (5) 6'-O-Benzoyl-4''-hydroxy-3''-methoxypaeoniflorin, (6) Arachidonic acid, (7) Bilirubin, (8) Heterodendrin, (9) Kaempferol-3-glucuronide, (10) Lotaustralin, (11) Morin, (12) Mudanpioside J, (13) Myricetin (14), Stearidonic acid. (**B**) ES negative: (1) (E,E)-9-Oxooctadeca-10,12- dienoic acid, (2) 1,2,3,4,6-Penta-O-galloyl-β-D-glucopyranoside, (3,4) 1,3-Dihydroxy-2-hydroxymethylanthraquinone-3-O- β-D -β-D-glucopyranoside, (5) 3,5,7,2′, 6′-Hydroxy-flavone 5-2′-O-β-D-glucopyranoside, (6,7) 6-Hydroxykaempferol-3-O- glucoside, (8,9) Cannabiscitrin, (10) Cimicifugic acid C, (11) Coronaric acid, (12) Isohyperoside, (13) Myricetin, (14) Myricetin-3-O-galactopyranoside, (15, 16, 17) Patuletin-7-O-[6′′-(2-methylbutyryl)]-glucoside, (18) Psamosilenins A, (19, 20) Quercetagetin, (21) Quercetin, (22) Quercimeritrin, (23) QuinquenosideI, (24) Sanleng acid.
